# The Use of Chitosan, Alginate, and Pectin in the Biomedical and Food Sector—Biocompatibility, Bioadhesiveness, and Biodegradability

**DOI:** 10.3390/polym11111837

**Published:** 2019-11-08

**Authors:** Gheorghe Adrian Martău, Mihaela Mihai, Dan Cristian Vodnar

**Affiliations:** 1 Faculty of Food Science and Technology, University of Agricultural Sciences and Veterinary Medicine, Calea Mănăştur 3–5, 400372 Cluj–Napoca, Romania; Adrian.martau@usamvcluj.ro (G.A.M.); mihaela.mihai@usamvcluj.ro (M.M.); 2 Institute of Life Sciences, University of Agricultural Sciences and Veterinary Medicine, Calea Mănăştur 3–5, 400372 Cluj–Napoca, Romania

**Keywords:** biopolymer, food industry, alginate, chitosan, pectin, biodegradability, biocompatibility, bioadhesiveness, limitation

## Abstract

Nowadays, biopolymers as intelligent and active biopolymer systems in the food and pharmaceutical industry are of considerable interest in their use. With this association in view, biopolymers such as chitosan, alginate, pectin, cellulose, agarose, guar gum, agar, carrageenan, gelatin, dextran, xanthan, and other polymers have received significant attention in recent years due to their abundance and natural availability. Furthermore, their versatile properties such as non-toxicity, biocompatibility, biodegradability, and flexibility offer significant functionalities with multifunctional applications. The purpose of this review is to summarize the most compatible biopolymers such as chitosan, alginate, and pectin, which are used for application in food, biotechnological processes, and biomedical applications. Therefore, chitosan, alginate, and pectin are biopolymers (used in the food industry as a stabilizing, thickening, capsular agent, and packaging) with great potential for future developments. Moreover, this review highlights their characteristics, with a particular focus on their potential for biocompatibility, biodegradability, bioadhesiveness, and their limitations on certain factors in the human gastrointestinal tract.

## 1. Introduction

Biopolymers are polymers obtained from natural sources, either entirely biosynthesized by living organisms or chemically synthesized from biological material [1,2,3]. These polymers are found in a multitude of food products and health maintenance products, which use biopolymers in the formulation as a functional excipient or as an active ingredient (active substance) [4,5,6]. At the same time, their diverse composition, physical behavior, and the wide variety of which to choose from, have fueled the interest in biopolymers. Moreover, their relatively low cost and renewable nature make this class of materials especially attractive to high-value sectors such as the food, biomedical, and pharmaceutical industries [7,8,9,10].

As such, the use of biopolymers from diversified sources has been studied for many years for food, biomedical, and pharmaceutical applications [11,12]. The global biopolymer market is expected to reach around 10 billion USD by 2021, increasing by almost 17% over the forecast period 2017–2021. Western Europe comprises the largest market segment, accounting for 41.5% of the global market [13]. This development is due to the increasing use of biopolymers. For example, biopolymers such as chitosan, alginate, and pectin can be used in the food industry in applications for food packaging [14,15,16], coating of fresh and cut fruits or vegetables [17,18,19], and for the pharmaceutical industry as microencapsulating agents or drug coatings [20,21,22,23,24]. These encapsulating agents have important roles in encapsulation efficiency and microparticle stability. Microencapsulation using biopolymers is considered to be a convenient protective method. Numerous food components have been successfully encapsulated, such as antioxidants [25], enzymes [26], vitamins [27,28], and minerals [29,30,31].

Renewable biomaterials are green options to reduce environmental pollution and waste formation [10,32]. In recent years, remarkable and creative ways of utilizing biopolymer-based materials have delivered a continuous development for sustainable bioeconomy and biotechnology [33,34,35]. A large number of recent articles have led to the expansion of evidence suggesting that enzyme-mediated catalytic bioprocesses have many advantages over conventional synthetic pathways and, therefore, they are increasingly important for multiple biotechnological applications, including biocatalysis, food products, environmental protection, biomedicine, bioenergy, biosensor development, and agro-chemistry based on renewable biomaterials [36,37,38,39,40,41,42].

There is a lot of confusion and too many definitions related to the terms “biopolymer”, “biodegradable”, “renewable resources”, etc. As such, biopolymers are polymers formed from natural sources or entirely biosynthesized by living organisms, and thus are biodegradable. IUPAC (International Union of Pure and Applied Chemists) defines biopolymers as a substances composed of a single type of biomacromolecule [43]. Obviously, the degradation of the polymers is represented by macromolecules that can undergo chain scissions, resulting in a decrease of molar mass [43]. Additionally, degradability in biopolymer materials is most often not determined by the origin of the raw materials used or by the process used for manufacturing these polymers, but may be influenced by the chemical and physical microstructure of the polymers [44]. Polymers from the natural category tend to be readily biodegradable, although the rate of degradation is generally inversely proportional to the extent of chemical modification [45]. However, not all natural polymers are strongly biodegradable (e.g., cellulose) and not all synthetic ones are environmentally stable [43,46,47]. For these reasons, Mensitieri et al. (2011) suggest that polymers removed or extracted from natural resources can be decomposed under different environmental conditions and under the action of different microorganisms [48].

**Figure 1:**
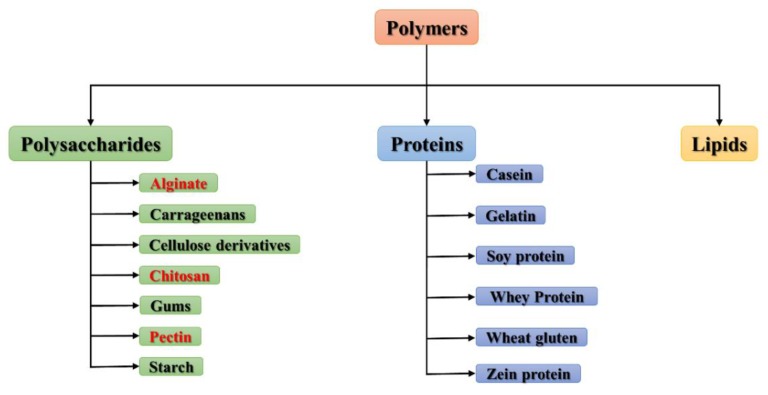
Classification of polymers from the nutritional point of view as carbohydrates, proteins, and lipids.

In his 2018 book, Tomy J. Gutierrez includes a classification of edible polymers from the nutritional point of view, namely carbohydrates, proteins, and lipids, i.e., they are considered as macronutrients (Figure 1) [49]. Also, some authors have classified polymers according to their production method or source as: Polymers extracted directly or removed from plant or animal biomass; polymers produced by classical chemical synthesis starting from bio-renewable monomers, such as polylactic acid (PLA); and polymers produced by microorganisms such as polyhydroxyalkanoates, cellulose, xanthan, and pullulan [48,50,51].

Chitosan (C_56_H_103_N_9_O_39_, M.W. 1526.5 g/mol), a polycationic polymer, is a non-toxic, biocompatible, and biodegradable biopolymer [52]. It has antimicrobial activity, observed in numerous studies, some of which have led to the creation of biodegradable labels. One such example is the label obtained with green tea extract and chitosan that has a decontamination effect on the surface of the studied fruits and vegetables. Other studies have shown its ability to extend the shelf-life of fruit products [53,54,55,56]. Chitosan also has a good mucoadhesive characteristic so that it is adsorbed to the mucous membrane along the gastrointestinal tract [57], and thus, a compatible carrier for colon-targeted probiotic microorganisms or drugs [58]. Nevertheless, chitosan may have a major disadvantage as it can be dissolved in an acid solution, which causes chitosan to lose its mucoadhesivity by deprotonation [59].

Alginate (C_12_H_20_O_12_P_2_, M.W. 418.23 g/mol), a natural polyanionic polymer, is a non-immunogenic, non-toxic, biodegradable polymer [60]. Alginates are widely used in a variety of applications, including applying coatings on fresh and cut fruits and vegetables [61,62], food protection[63], as thickening, gelling, emulsifying, and stabilizing agents in food products such as ice cream, sauces, and fruit pies [64], and drug delivery systems for anti-reflux preparations [65]. Alginate has antioxidative and anti-inflammatory properties and it is stable in the stomach acidic gastric solution and can gradually dissolve under alkaline conditions in the small intestine [66,67,68]. In the rational drug design for chemotherapy, alginate shows an application in tumor therapy [69].

Pectin (C_6_H_10_O_7_, M.W. 194.14 g/mol), an anionic biopolymer, is another important polymer used in the food industry. It is soluble in water and it is one of the major structural polysaccharides of higher plant cells. Pectin has many applications in the food and beverage industry as a thickening and gelling agent, colloidal stabilizer, texturizer, and emulsifier [70,71], and for applying coatings on fresh and cut fruits or vegetables [72,73]. Pectin is generally formed of water-soluble pectinic acids with varying methyl ester contents, which are capable of forming gels alongside sugar and acid when exposed to the correct conditions [74]. Pectin for food technology or pharmaceuticals, especially for colon treatments, has been comprehensively reviewed over time [75,76,77,78]. Pectin is a high molecular weight branched macromolecule, which can be transformed into a hydrogel, intended as a flexible network of polymeric chains that can swell but not dissolve in water [79].

This review discusses the crucial parameters for the most relevant biopolymers: A polycationic polymer—chitosan; a polyanionic polymer—alginate; and an anionic biopolymer—pectin. The objectives considered in the review are the use of biopolymers in the food industry, biotechnological processes, and biomedical applications, with a main focus on the use as stabilizing, thickening, or capsular agents, as well as for packaging. These biopolymers are considered to be polymers with great potential for future developments. This review particularly highlights their characteristics, with emphasis on their potential for biocompatibility, biodegradability, and bioadhesiveness, alongside their limitations under certain conditions, such as those from the human gastrointestinal tract, following consumption.

## 2. Chitosan

### 2.1. History, Structure, and Sources

European authorities approved chitosan as safe for consumption, and a monograph on chitosan hydrochloride was included in the fourth edition of the European Pharmacopoeia (2002). In addition, it is an approved food additive in Japan and has been widely used in the food industry.

**Figure 2:**
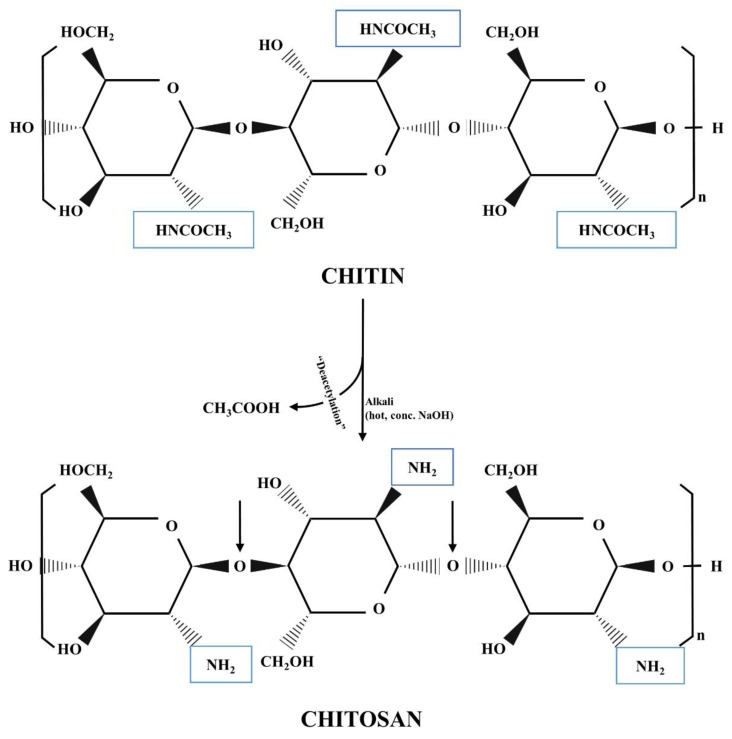
Chemical structure of chitin and chitosan, chitosan production from chitin.

Chitosan is a partly deacetylated polymer of N–acetyl glucosamine. It is a natural, water-soluble derivative of chitin with distinctive properties. Chitosan is generally prepared from chitin (Figure 2) (2 acetamido–2–deoxy–1,4–D–glucan) and chitin may be found in a lot of natural sources [80,81]. Chitin is the second most abundant biopolymer in nature after cellulose [8,82]. Chitosan (1 →4)–linked 2–amino–2–deoxy–b–D–glucan, can be obtained from chitin through alkaline hydrolysis of the N–acetyl groups. Upon further hydrolysis, for example, with the help of chitosanases, indicated by black arrows, low molecular weight (MW) oligosaccharides are produced.

Chitin is the primary structural component of the outer skeletons of crustaceans and is also found in many other species such as mollusks, insects, and fungi [83]. The most frequently obtained form of chitosan is the α–chitosan from crustaceans, namely chitin from shrimp shell and crab shell wastes [83]. Chitin accounts for around 70% of the organic compounds in such shells. In the process of obtaining chitosan, ground shells are demineralized and deproteinized by sequential treatment with acid and alkali, after which the extracted chitin is deacetylated to chitosan by alkaline hydrolysis at high temperature. Production of chitosan from these sources is cheap and straightforward. It has also been suggested that other sources of chitin, e.g., β–chitin from squid pens, may be valuable in relation to the preparation of chitosan [84,85]. Chitosan in nature as such is rare, except in certain fungi. In recent years, the production of chitosan from fungi, using fermentation methods, has also gained much interest [86]. The physicochemical characteristics of chitosan are closely related to the taxonomy of the source [84].

### 2.2. Properties and Applications of Chitosan

Chitosan is widely used in a range of diverse fields, including food, agriculture, waste management, and medicine. Obviously, chitosan as a composite material has been extensively studied. Its various properties (antimicrobial properties; permeability and solubility; it decreases swelling and improves mechanical properties), make it very suitable for possible future applications in food and drug packaging, antimicrobial films, and coatings, such as applying coatings on fresh and cut fruits or vegetables (Table 1).

#### 2.2.1. Biocompatibility and Biodegradability

Chitosan can play an important role in regulating growth and eliciting defense in many plant species. However, the exact metabolic response of plants to chitosan is still not clear [87]. The effect of the degree of deacetylation of chitosan on properties such as solubility and antimicrobial activity has been studied in a multitude of articles [88,89]. Additionally, chitosan is a potentially useful pharmaceutical material owing to its low toxicity and good biocompatibility [90,91].

It has also been marketed throughout the world as a component in non-medical products, as a fat binder in cholesterol-lowering and slimming formulations [92]. Thus, it has been observed that chitosan entraps in lipids in the intestine, because of its cationic nature [85,93]. Analyses performed in the biomedical field have revealed it to be highly biocompatible [90,94]. At the same time, chitosan is metabolized by certain human enzymes, especially lysozyme, and is considered biodegradable [91]. Chitosan has unique biological properties such as biocompatibility, biodegradability, and mucoadhesion. It is also anticholesterolemic, antimicrobial, and exhibits permeation enhancement effects. These properties have led to its increased utilization in distinct applications such as antibacterial/anti-biofouling coatings, controlled release coatings and microcapsules, nanofiltration, drug delivery hydrogels, gene delivery, and tissue engineering scaffolds. For biomedical applications, aiming to reach in vivo testing, chitosan derived from non-animal origins is preferred [95,96,97,98].

#### 2.2.2. Bioadhesiveness

One area of growing interest is the use of chitosan as a bioadhesive material. Many commercially available chitosans exhibit fairly good mucoadhesive properties in vitro [99]. The mucoadhesive properties of chitosan have been illustrated by its ability to adhere to porcine gastric mucosa in vitro [100], and may therefore be useful for the administration of site-specific drugs. It has been suggested that residence time of formulations at sites of drug action or absorption could be prolonged with chitosan. It has also been recommended that chitosan might be valuable for delivery of vitamins, minerals, or other drugs to specific regions of the gastrointestinal tract like the stomach [100,101], small intestine [99,102,103], and buccal mucosa [104,105]. The adhesive properties of chitosan in a swollen state have been shown to persist well during repeated contacts of chitosan and the substrate [99], which implies that, in addition to the adhesion by hydration, many other mechanisms, such as hydrogen bonding and ionic interactions, might also have been involved. A very important mechanism of action was recommended to be the ionic interaction between the negatively charged mucus gel layer and the positively charged amino groups in chitosan. In the interactions between chitosan and mucus [102,106], the primary mechanism of action at the molecular level was found to be electrostatic [107]. The interactions are strong at acidic and slightly acidic pH levels, where the charge density of chitosan is high [102]. Growth in molecular weight of chitosan results in stronger adhesion [99].

It was shown that the amounts of chitosan microspheres adhering to the intestine were greatest when the density of cross-linking of chitosan was lower (i.e., when the number of free amino groups in chitosan was higher) [102]. This also suggests that the adhesive properties of chitosan should increase as the degree of deacetylation increases, while cross-linking reduces the mucoadhesive effects of chitosan [108,109].

#### 2.2.3. Chitosan Absorption

In recent years, chitosan has attracted much attention as a potential absorption enhancer across mucosal epithelia, especially for peptide drugs [120,121,122]. It has been revealed that chitosan acts as a permeation enhancer by opening epithelial tight junctions [123]. In 1994, Illum et al. showed the permeation enhancing capabilities of chitosan for the first time [120]. Chitosan has the ability to enhance the paracellular route of absorption, which is very important for the transport of hydrophilic compounds such as therapeutic peptides and antisense oligonucleotides across the membrane. The mechanism underlying this permeability enhancement effect appears to be based on the positive charges of the polymer, which interacts with the cell membrane resulting in a structural reorganization of the proteins associated with tight junctions [124]. As such, chitosan has some advantages over small molecular weight-enhancing agents. The mucoadhesive properties allow it to remain concentrated in the absorption zone of the drug [125]. Additionally, the ability of chitosan to act as an absorption enhancer has been demonstrated in Caco–2 cells, which serve as a model of intestinal epithelium [126,127,128,129], as well as in vitro experiments on nasal, buccal, vaginal, and urinary bladder mucosa of different animals [130,131,132,133,134]. Chitosan also increased the bioavailability of a peptide drug, which was applied during intraduodenal in vivo experiments in rats [106]. Moreover, it has been suggested that chitosan may reduce the apparent digestible fats by the following procedure: The consumed chitosan is dissolved in the stomach gastric acid and the dissolved chitosan is mixed with dietary fat to form complex chitosan fat. This process subsequently forms complex gels in the small intestine; and dietary fat alongside the gel is excreted in the feces [93].

#### 2.2.4. pH Sensitiveness

Chitosan exhibits a pH-sensitive behavior as a weak base due to the large amounts of amino groups in its chain. Chitosan is insoluble at higher pH ranges while it dissolves easily at low pH. Under low pH conditions, the sensitive mechanism swelling involves the protonation of amine groups of chitosan [135]. This property has helped chitosan to be used in the delivery of chemical drugs to the stomach and has been widely investigated as a delivery matrix. Nevertheless, for the delivery of protein drugs to the intestine, this property has a limitation; as the matrix is dissolved in the stomach, the released protein drugs or other compounds of interest will get denatured. Moreover, the pH sensitivity of the native chitosan is not suitable for protein delivery. To overcome this, many changes can be made to improve the stability of chitosan in the stomach and the subsequent controlled delivery of protein drugs in the intestine [94].

### 2.3. Limitations

The main limitation of chitosan for the administration of different compounds or different drugs is the easy dissolution of chitosan in the low pH of the stomach. Nonetheless, the favorable properties of chitosan, such as improved absorption and mucoadhesiveness, have been shown to occur in low pH conditions. This is because, as a weak base, chitosan requires a certain amount of acid to convert the glucosamine units into the positively charged water-soluble form [136]. The poor solubility of chitosan represents a barrier for it to perform its mucoadhesive and absorption enhancing properties in the small intestine, which is the main absorptive region of the gastrointestinal (GI) tract. Therefore, to make it a suitable matrix for the administration of some proteins, several chemical modifications are necessary. The different chitosan derivatives with favorable properties have been developed and found to serve this purpose, with improved functionality also at higher pH levels [94].

## 3. Alginate

### 3.1. History, Structure, and Sources

**Figure 3:**
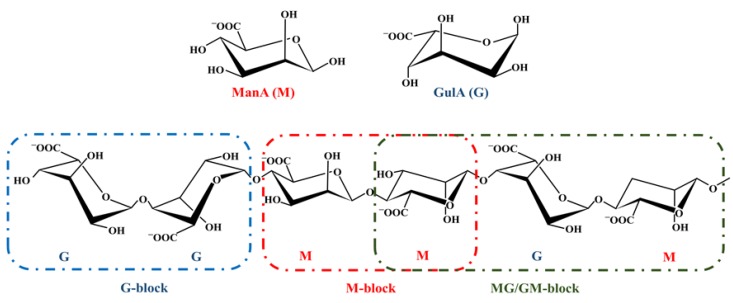
Chemical structure of alginate. The alginate monomers β–D–mannuronic acid (ManA; M) and α–L–guluronic acid (GulA; G), as well as an alginate chain illustrating linkage conformation and block composition.

Alginate was first isolated by Stanford in 1881 [137] and has since become a multifunctional ingredient in many applications. Alginates are included in a group of compounds that are generally considered safe by the FDA. Generally, alginates are assigned their special role in wound healing. The regular use of alginates as dressings for wounds dates back to the early 1980s when several lined products became commercially available. Alginate (Figure 3) is a water-soluble polysaccharide composed of alternative blocks of 1–4 linked α–L–guluronic acid (GulA; G) and β–D–mannuronic acid (ManA; M) residues [94]. Alginates may contain G-blocks, M-blocks, and/or MG/GM-blocks of varying lengths (Figure 3). Alginate is obtained from several different species of brown seaweed (*Phaeophyceae*) and is present as sodium, magnesium, and calcium salts of alginic acid. The species that are generally used for the production of commercial alginates are *Macrocystis pyrifera*, *Laminaria hyperborea*, *Saccharina japonica*, and *Ascophyllum nodosum* [138] wherein the alginate may comprise up to 40% of the dry weight [139,140]. In addition to seaweed, alginate can be synthesized by several species of bacteria; bacterial alginates were isolated from *Azotobacter vinelandii* and several *Pseudomonas* species [141]. However, alginate from a bacterial source is not yet commercially available [142]. The process of extraction of alginate has been well-analyzed in literature (e.g., Nussinovitch, 1997 [143]) and is relatively simple. The first step in obtaining alginate is for the raw material from the algae to be ground and washed with acid before extraction with hot alkali. The second step is for the extract to be filtered, precipitated with calcium, and acidified to produce alginic acid. The insoluble alginic acid can then be treated with metallic carbonate, hydroxide, or oxide to produce the desired salt form of alginate [144].

### 3.2. Properties and Applications of Alginate

Alginate is widely used in a range of diverse fields, including food, agriculture, and medicine. Obviously, alginate as a composite material has been widely studied. It has various properties (antimicrobial and antiviral properties; permeability and solubility; decreases water solubility; and it improves mechanical properties), making it very suitable for possible future applications in food and drug packaging, antimicrobial films, and coatings, for example applying coatings on fresh and cut fruits or vegetables (Table 2).

#### 3.2.1. Biocompatibility and Biodegradability

Alginate is used broadly in the food industry as a thickener, emulsifier, and stabilizer. The oral administration of alginate has not been shown to provoke many immune responses unlike the intravenously administered forms and it is reported that alginate is non-toxic and biodegradable when given orally [145]. Although alginate biocompatibility has been extensively investigated, there is a disagreement in literature. In the case of intravenous administration, the induction of foreign body reaction and fibrosis have been reported for most commercial alginates [146,147], while other reports show little or no immune response around alginate implants [148]. Usually, alginates are available when tested after purification by free-flow electrophoresis and do not provoke foreign body reactions, at least three weeks after implantation in the peritoneal cavity of rodents [149]. Immunogenic response to intravenous injections may be due to toxic contaminants from commercial alginates [94].

#### 3.2.2. Bioadhesiveness

Mucoadhesive microorganisms or drug delivery systems work by increasing the residence time at the site of activity or resorption. The mucoadhesive feature of alginate may help in its usefulness as a potential prebiotic, probiotic bacteria, or drug delivery vehicle in mucosal tissues, such as the GI tract [150]. Studies have shown that polymers with charge density can serve as good mucoadhesive agents [151,152,153]. It has also been reported that polyanionic polymers are more effective as bioadhesives than polycation polymers or nonionic polymers [151]. Alginate, being an anionic polymer with carboxylic groups, is therefore a good mucoadhesive agent. Studies have shown that alginate has the highest mucoadhesive strength compared to polymers such as chitosan, carboxymethyl cellulose, or polylactic acid [152]. Due to the adhesion of alginate particles to the mucosal tissues, the transit time of the protein is delayed, and probiotic microorganisms or a certain drug may be located on the absorbent surfaces. This can improve the bioavailability and effectiveness of probiotics or drugs [94].

#### 3.2.3. Alginate Absorption

Previous in vitro results indicated that alginate beads might be a useful vehicle for iron fortifying foods. A human study was undertaken to test the hypothesis that alginate enhances iron absorption. A single blind, randomized crossover study was conducted to measure serum iron absorption after a test meal. The conclusion of this study shows that alginate beads are not a useful system for releasing soluble iron salts for food fortification [154].

Natural alginate fiber may be used as templates for the manufacture of hierarchically porous carbon fiber decorated in CoFe alloy [155]. In addition, in 19 human subjects, the effect of sodium alginate on GI uptake of strontium and calcium was investigated. Fifteen volunteers were given 1.5 g of alginate, two were given 3.0 g, and two 0.3 g. The group with 1.5 g of alginate reduced strontium absorption by a factor of two, without significant results on calcium absorption. The lower dose of alginate with 0.3 g appeared to have no effect on strontium or calcium absorption, and the higher dose with 3.0 g did not have an effect greater than the 1.5 g dose [156]. Different studies evaluated the effects of alginate oligosaccharide supplementation (AOS) [157,158,159]. Besides these studies, another study transmitted two trials to assess the effects of AOS supplementation on the growth performance, antioxidant capacity, serum hormone levels, and intestinal digestion-absorption function in weaned pigs [160]. Presently, many oligosaccharides have shown beneficial effects in mitigating physiological disorders after weaning [160,161,162].

#### 3.2.4. pH Sensitivity

The release of macromolecules from alginate in low pH solutions is significantly reduced, which could be advantageous in developing a system for oral administration of certain compounds. [163,164]. Apparently, alginate shrinks at low pH (gastric environment) and the encapsulated prebiotic, probiotic microorganisms, or different drugs are not released [165]. In the gastric tract, the hydrated sodium alginate is transformed into a porous, insoluble layer of alginic acid. Once passed into the higher pH of the intestinal tract, the alginic acid layer is converted into a soluble viscous layer. This pH condition of alginate can be exploited to customize release profiles. Nonetheless, the rapid dissolution of alginate matrices in higher pH ranges can lead to release by an explosion, which is not desirable for protein drugs because this causes protein drugs to be denatured by proteolytic enzymes. However, many changes in physicochemical properties are required for prolonged controlled release of protein drugs [94].

### 3.3. Limitations

**Figure 4:**
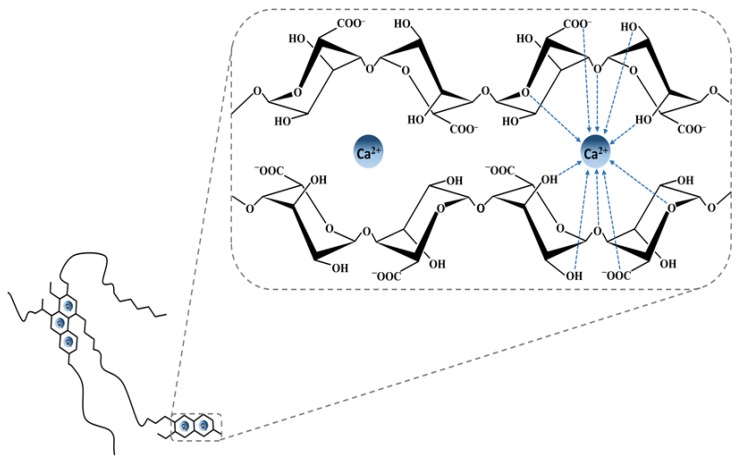
Model describing the interactions between alginate G-blocks and divalent cations (Ca^2+^), which results in ionic gel formation.

A significant problem arises in the preparation of calcium alginate. Despite the fact that calcium alginate (Figure 4) can be prepared by simple and easy procedures, this method has a major limitation, namely the loss of the compound during preparation by bonding the created pores [174,175]. In this case, many alginate modifications have been tested for the administration of compounds such as drugs, some with success and others with failure. The crosslinking of alginates with aldehydes has been done successfully. Sodium alginate alone [176,177,178] or together with gelatin or ovalbumin [179] were cross-linked with aldehydes, and their microparticles and beads were prepared for various applications. The cross-linked alginate has more capacity to retain trapped compounds and has a more controlled release profile of the compound or drug entrapped. In 2002, Chan et al. [176] proposed that pentane diol with two aldehyde groups can produce cross-linkage between two alginate molecules through formation between two hydroxyl groups via pentanedial. Many other methods have been adopted by some researchers to overcome the limitations presented by the relatively large pore size and the physical instability of alginate in higher pH environments. In addition, the changes made alginate hydrogels exhibit some additional improved features to help perform their task of protein delivery more efficiently [94].

## 4. Pectins

### 4.1. History, Structure, and Sources

Henri Braconnot first isolated pectin in 1825, although the action of pectin to make jams was well known [180,181]. In the joint FAO/WHO expert report on food additives and in the European Union, no acceptable daily intake has been established as pectin is considered safe [182].

**Figure 5:**
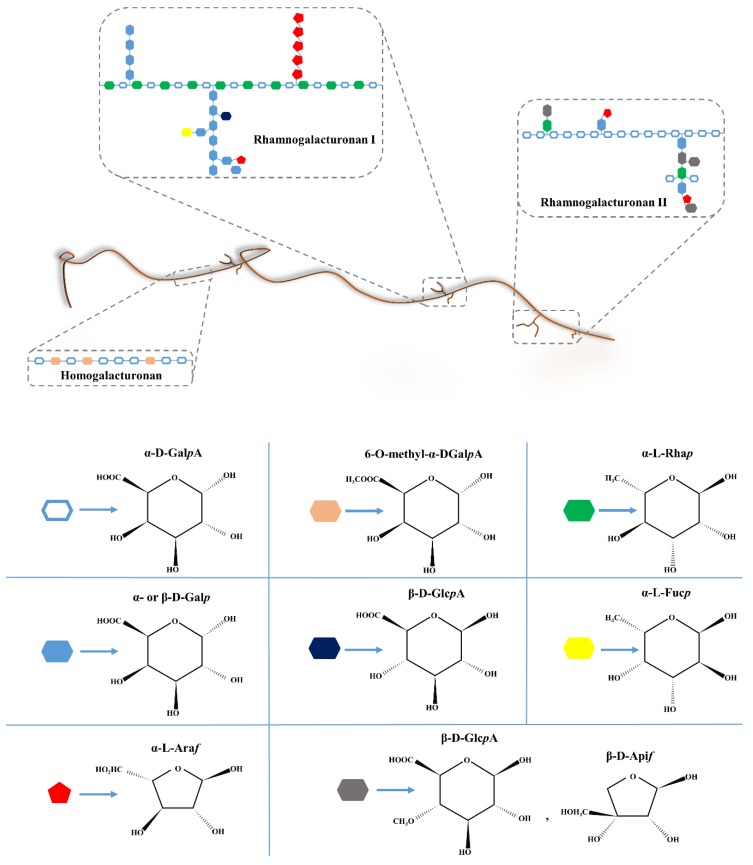
Schematic representation of the pectin structure with the main domains and monosaccharide composition, adapted from References [78,183,184,185].

The structure of the pectin can greatly affect the properties of the gels: The monosaccharide content, the branching, and the spatial arrangement of the cross-linking blocks must be carefully considered when designing the pectin gels for specific biomedical applications. As for many other naturally occurring polymers, the molecular weight of pectin, the degree of esterification (DE), and acetyl esterification are heterogeneous, depending on the source and the conditions of pectin extraction. Pectin is composed of at least three types of polysaccharides: Rhamnogalacturonan–I (RG–I), Rhamnogalacturonan–II (RG–II), and Homogalacturonan (HGA) (Figure 5). HGA is the principal component of pectin, and contains α–(1 →4)–D–linked galacturonic acids (1,4–α–D–Gal*p*A) that are partially methyl-esterified and occasionally acetyl-esterified [8,78].

Methyl-esterified residues (6–O–methyl–α–D–Gal*p*A) distribution of the HGA backbone to the total carboxylic acid units in the salt form represents DE [186]. After DE, pectins are classified as low methoxyl (LM), (DE < 50%) or high methoxyl (HM), (DE > 50%), each having different properties. The properties have been found to profoundly affect the properties of the gels formed and, therefore, need to be carefully controlled according to the need for application [187]. RG–I is represented by the disaccharide unit galacturonic acid–rhamnose (1,4–α–D–Gal*p*A–1,2–α–l–Rha*p*–)_n_, approximately 20–80% of the Rha*p* residues being substituted with neutral oligosaccharides, mainly arabinofuranose and galactose (α–l–Ara*f* and β–D–Gal*p*) (Figure 4). Moreover, glucopyranose and 4–O–methylglucopyranose (α–L–Fuc*p*, β–D–Glc*p*A) can be found as terminal residues of the side chains (Figure 4). RG–II represents a more complex structure: It presents a backbone consisting of 1,4–α–D–Gal*p*A, and side chains of different sugars such as rhamnose, galacturonic acid, galactose, arabinofuranose, fucose, apiofuranose (α–l–Rha*p*, α–D–Gal*p*A, α– or β–D–Gal*p*, α–l–Ara*f*, α–l–Fuc*p*, β–D–Api*f*), and more. One hypothesis concerns a model in which the HG, RG–I, and RG–II backbones are covalently cross-linked to form block co-polymers, but the relative position of the three main areas is not yet fully known. The alternative regions are, in fact, the traditional model to describe the disposition of the domains: It is formed from one linear backbone of the unbranched HGA residues alternately linked to branched RG–I residues [184,188]. Nevertheless, recent studies on pectin composition reported other possible models for pectin structure: The RG–I backbone model, in which HGA is positioned as a RG–I side chain [189], in which unsubstituted HGA is connected with RG–I but with no linear configurations [183].

Pectin is one of the main constituents of citrus fruits, apple, and mango, and has good gelling properties [9,74]. Pectin can be extracted from many food industry by-products, such as fruit and vegetable pomaces. A lot of residues result from the extraction of sugar, the most relevant being the sugar beet pulp, which is a rich source of pectin [70]. Pectins are a complex family of heteropolysaccharides that make up a large part of the primary cell walls of dicotyledonous plants and play important roles in their growth and development [185,190].

### 4.2. Properties and Applications of Pectin

Pectin is widely studied in a range of diverse fields, including food, agriculture, and medicine. Obviously, pectin as a composite material has been widely studied, exhibiting various properties (antimicrobial and antiviral properties; it decreases water solubility; and improves mechanical properties), making it very suitable for possible future applications in food and drug packaging, antimicrobial films, and coatings, such as applying coatings on fresh and cut fruits or vegetables (Table 3). Additionally, pectins have been extensively studied in the pharmaceutical industry for drug administration, wound dressing, and tissue engineering. Pectins have shown many advantages in these formulations, as they can be easily adapted to hydrogels, films, scaffolds, and nanoparticles [78,191,192,193].

#### 4.2.1. Biocompatibility and Biodegradability

Oral delivery is still the preferred route of administration of various compounds, especially medicines, for chronic pathologies in which repeated administration is required. Researchers have long used pectin as a potential carrier of drugs or other compounds of interest for the colon [194,195]. At the same time, different compounds (drugs) are transported over long distances and exhibit different environmental conditions, such as low pH and mechanical pressure in the stomach, protease attack in the small intestine, and digestion of microflora in the colon [196]. For these reasons, oral administration of compounds is not suitable for the administration of most proteins and polypeptide compounds, due to their high susceptibility to digestive enzymes in the gastrointestinal tract, poor absorption, and limited ability to transport across intestinal barriers [77].

Hydrophilic polymeric matrix systems are widely used in the oral administration of controlled compounds, due to their flexibility to obtain a compound release profile, profitability, and wide acceptance of the regulations [187,197,198]. The ability of hydrophilic polymer matrices to release a compound trapped in an aqueous environment and to regulate the release of such a compound by controlling swelling and cross-linking makes them particularly suitable for controlled release applications [197]. Lately, many controlled release formulations based on hydrophilic polymeric matrices have been developed [198,199,200].

A recent interest that has developed in the commercial use of pectins is wound healing. This is partly due to their long-standing reputation for being non-toxic or generally considered safe [77,196,201], with relatively low production costs [187] and high availability [202]. Moreover, because the gelling mechanisms are relatively simple, there is an interest in the preparation of hydrogels for biomedical applications, such as drug administration, gene delivery, and wound healing [78].

#### 4.2.2. Bioadhesiveness

The release pattern of the compounds can be constant, oscillating, continuously decreasing, or even pulsatile. For most drug delivery systems, natural polymers are used as harmless and biocompatible carriers [203,204]. Among natural polymers, pectin has interesting properties for administration applications for different compounds or drugs, such as mucosal adhesion, ease of dissolution in basic media, and the ability to form gels in acidic media. Its muco-adhesiveness can be exploited to target and control the administration of some compounds in the nasal or gastric environment, while the ease of dissolution in the basic media, together with its resistance to proteases and amylases, makes pectin suitable for drug delivery in the colon.

The ability to form gels under acidic conditions improves the contact time of compounds (drugs) for gastric or ocular treatments [205,206]. However, pectin has been found to recognize galectin molecules, which are involved in various stages of cancer pathologies, being particularly attractive to target tumor cells for chemotherapy treatments [207]. From a biomedical perspective, understanding the organization of pectin domains can be fundamental for adapting cell adhesion and mucoadhesive or anti-metastatic properties of pectin gels and for the formation of mechanically stable gels.

#### 4.2.3. Pectin Absorption

To protect various compounds against degradation and to achieve targeted release in certain organs, the compounds (drugs) are encapsulated in micro- or nano-capsules. Pectins were considered in the preparation of capsules for the sustainable administration of different compounds and for masking the taste. The ability of pectins to be resistant to proteases and amylases, which are active in the gastrointestinal tract, and to be degraded by the intestinal microflora make them suitable for colon medications, proteins, or polypeptide administration [199]. As a disadvantage, pectin gels are swollen in aqueous media and a small amount of compound (drug) can be released into the GI tract. To avoid this problem, divalent ions such as Ca^2+^, Zn^2+^ or other polymers such as chitosan, ethylcellulose, or hydroxypropylmethyl cellulose [77,187,208,209,210,211], have been used to form strong pectin gels, for the administration of various compounds or even medicines in the colon. The ability of pectin gels to swell under acidic conditions can be considered a real advantage if these systems considered are used for weight reduction and obesity treatments. In fact, when pectin gels reach the aqueous environment of gastric fluids, the gels begin to swell, thus filling the stomach and adhering to the stomach walls long before digestion, leading to a prolonged non-appetite sensation [212].

In a 2007 study, Sungthongjeen et al. [200] researched the effects of compression force, ratio of drug to pectin, and grade of HM pectin on drug release from matrix tablets. The release of the compound from the matrix tablets could be altered by the degree of pectin HM and the ratio between the compound and pectin. The DE, which is an important characteristic of pectin and may influence the release of a compound from the system, has not yet been thoroughly examined [187].

#### 4.2.4. pH Sensitivity

HM pectins (with DE > 50%) require a relatively high concentration of soluble solids and a low pH for gel formation [214,215]. LM pectins (with DE < 50%) form rigid gels by the action of calcium or multivalent cations, which cross-link the galacturonic acid chains [214]. Non-toxicity and low production costs of pectins are of particular interest in formulating controlled release dosage forms [199,200].

Citrus pectin modified by high pH and temperature treatments [207,216] was used to target galectin–3 (Gal3). Pectin appears to be able to inhibit cancer metastasis and primary tumor growth in several cancers in animals [207,217,218,219]. It has been suggested that the inhibitory effect is due to the recognition of galactan components of pectin by Gal3. Thus, modified pectins, possibly loaded with cytotoxic drugs to induce apoptosis of neoplastic cells, have the potential to dramatically increase the efficiency of conventional chemotherapy [78]. The suggested role of modified citrus pectin as a galectin–3 inhibitor is described as an established fact, but it is not. The assumption from previous publications that modified citrus pectin is a good specific inhibitor of galectin–3 contravenes other articles [220,221]. Instead modified citrus pectin and other plant polysaccharides may have other effects unrelated to galectin–3, as found e.g., in cells not expressing galectin–3 [222].

Another study optimized the effects of processing variables for obtaining pectin from artichokes (pH, extraction temperature, extraction time). Thus, it was observed that pH can influence the process of obtaining pectin and optimum extraction conditions were represented at pH 1.52, 63.62 min, and 100 °C with a maximum pectin yield of 18.76% [223,224].

### 4.3. Limitations

Pectins work well with foods with low humidity, but contrary to this use, they may present as poor moisture barriers. Currently, one limitation is the existence of very little information on the application of edible films of pectin on meat foods [50]. Pectin films have low thermal stability and poor mechanical properties, which is why they have been mixed with different polymers to improve thermal and mechanical stability [225].

## 5. Perspectives and Future Trends

Life as we know it requires three basic types of polymers: Polypeptides, polynucleotides, and polysaccharides. Over time, biopolymers have become increasingly important and popular for research in the food and pharmaceutical industry or for various biomedical applications. Although their production and use are constantly increasing, many problems remain unknown and need to be fully analyzed and resolved, such as stability, optimal molar mass, and interactions with other compounds. At the same time, the multifunctional behavior and the stability of the biopolymers at different physical states facilitate their application in a wide variety of domains, from woven fibers that swell in contact with water, to hydrogels rich in water, which can encourage and maintain a humid environment. Due to the wide variety of biopolymers to choose from and the ability to mix biopolymers, there is an endless series of physical behaviors that can be designed for specific functionalities. Physiological compatibility and the ability to load and control the release of various compounds or drugs when exposed to different biological environments means that intelligent biopolymers that are physiologically responsive can be developed. The demand for products is likely to increase significantly over the next decades with an aging population. In particular, there is an increased demand for a renewable approach, which includes both medicines and different cell healing proteins. Moreover, advances in genomics, proteomics, and stem cell technology have increased the desire for more personalized therapies that are formulated based on knowledge of the patient’s individual biology. This has the potential to revolutionize wound healing treatments, and the many advantageous properties of biopolymers are likely to be used in the development and delivery of these treatments in the future.

Although the types of biopolymers are traditionally studied and learned in isolation, we believe polysaccharides are best understood in the context of common attributes and their key differences. Recognition of the universality of the biopolymer explains their structures and functions and indicates their origins. Only by examining biopolymers in context, we can hope to gain a reasonable understanding of the fundamental molecules of life.

Chitosan, alginate, and pectin are natural polysaccharides that are considered safe for human consumption and have been used for many years with great success both in the food and beverage industry as thickening agents, gelling agents, and colloidal stabilizers. They are also used in increasingly wider applications such as in the pharmaceutical industry and in biotechnology. The cross-linking properties of biopolymers with other material composites allowed them to be used as a matrix or membrane for antimicrobial film and coatings or the administration of a variety of compounds. In the future, broad development is expected in the biopolymer industry. Their findings and implications should solve humanity’s biggest problems in the broadest possible context. Future research directions will also be based on biopolymers, biotechnologies, and renewable sources.

## 6. Conclusions

Natural polymers play a very important role in our lives, sometimes visible and sometimes invisible. Researchers and scientists have achieved great success in developing new systems, from the development of biodegradable films to nanotechnology and smart packaging with biopolymers. On the other hand, natural polymers have received much more attention in recent decades due to their potential applications in the fields related to the food industry, the pharmaceutical industry, and the biomedical industry, but also for applications in maintaining physical health, so we can say that biopolymers represent a highly debated topic. Biocompatibility, biodegradability, bioadhesion, absorption, and limitations were the main important features analyzed in this review of chitosan, alginate, and pectin. These characteristics have been described to highlight the properties that can affect the formation of gels and, in particular, their absorption in the human body. The wide variety of current applications, as well as the increasing number of studies related to future applications, suggest that their potential for highly versatile biopolymers will be even more significant in the future.

## Figures and Tables

**Table 1:** Chitosan properties used for different applications.

Composite Material	Effect	Possible Application	Reference
Chitin nanocrystals	- Improve mechanical properties and transparency.	Packaging and food packaging.	[110]
Chitosan/MgO	- Improves mechanical properties; - Increases opacity; - Decreases swelling, permeability, and solubility; - Antimicrobial properties.	Food active packaging.	[16]
Chitosan with additional compounds of Propionic acid	- Propionic acid incorporation into chitosan films inhibits *Candida spp* and *Penicillium spp* growth; - Extended food shelf life by maintaining microbial growth in the latency period.	Antimicrobial films and coatings.	[111]
Chitosan with additional compounds of Microemulsions formed from C–BF–G as emulsifier additive with AIT and LAE as antimicrobials	- Micro emulsions create micro pores and micro channels that hold antimicrobials effectively; - Facilitates antimicrobial release from the center to the surface of films or coatings, thus enhancing their antimicrobial efficacy; - Films with 1% AIT reduced *Listeria innocua* populations in ready-to-eat meat and strawberries; - Films with 1% LAE reduced *Escherichia coli* and *Salmonella spp*. populations in strawberries.	Antimicrobial films and coatings.	[55]
Chitosan with additional compounds of PA	- Chitosan/PA composite films present more TPC and AA than chitosan films.	Antimicrobial films and coatings.	[112]
Chitosan with additional compounds of Hydroxybenzoic acids: GLA, GTA, PA, SA, and VA	- AA assays show that chitosan films with hydroxybenzoic acid have higher DPPH scavenging activity than films consisting of chitosan only; - GLA provides higher antioxidant activity.	Antimicrobial films and coatings.	[113]
Chitosan with additional compounds of *Cymbopogon citratus* (lemongrass) essential oil	- Coating decreases the severity of *Rhizopus soft rot*; - More significantly delays the infection when the fruit were artificially contaminated after coating application; - The application of the coating preserves the general quality of tomato fruit.	Applying coatings on fresh and cut fruits and vegetables.	[56]
Chitosan with additional compounds of Natamycin, nisin, pomegranate, and grape seed extract	- Coating reduces the O_2_ consumption of the fruit; - Shows better effects on delaying changes of pH, water activity, and TMC; - The incorporation of different antimicrobial agents into chitosan matrix does not reveal any significant effect.	Applying coatings on fresh and cut fruits and vegetables.	[114]
Chitosan with additional compounds of *Salvia fruticosa* Mill. extract	- The efficacy of the coating against grey mold is statistically equal to the synthetic fungicide thiabendazole; - Coating decreases the rate of fruit WL during cold storage, while preserved; - Coatings do not affect quality attributes and the bioactive compounds in table grapes.	Applying coatings on fresh and cut fruits and vegetables.	[19]
Chitosan with additional compounds of thyme essential oil nanoparticles	- The coating reduces the incidence of *C. gloeosporioides* on avocado; - Coating does not affect the quality of avocado; - Fruit is better maintained than untreated fruit.	Applying coatings on fresh and cut fruits and vegetables.	[115]
Chitosan with GP	- Casting method and film physical form.	Antimicrobial films and coatings.	[116]
Chitosan with FAA	- Coating physical form.	Oil barrier packaging.	[117]
Chitosan with additional compounds of Lemongrass oil	- Coating with nanodroplet of oil shows higher initial inhibition of *Salmonella typhimurium*; - Greater growth inhibition of microorganisms and higher retention of color; - AA and better SE during storage.	Applying coatings on fresh and cut fruits and vegetables.	[118]
Chitosan with GP and GTE	- Casting method and film physical form.	Active food packaging.	[119]
Edible polymers pectin–fish gelatin with glycerol plasticizer and Glutaraldehyde additives	- Casting method and film physical form.	Packaging or coating of food or drugs.	[23]

C–BF–G—corn–bio–fiber gum; AIT—allyl isothiocyanate; LAE—lauric arginate ester; PA—protocatechuic acid; TPC—total phenolic content; AA—antioxidant activity; GLA—gallic acid; GTA—gentisic acid; SA—syringic acid; VA—vanillic acid; DPPH—2,2-diphenyl–1–picrylhydrazyl; TMC—total microbial count; WL—weight loss; GP—glycerol plasticizer; FAA—fatty acid additives; GTE—green tea extract; SE—sensory evaluation.

**Table 2:** Alginate properties used for different applications.

Composite Material	Effect	Possible Application	Reference
Alginate with additional compounds of Ag nanoparticles	- Provide antimicrobial and antiviral properties.	Fresh food packaging, packaging for agricultural products.	[166,167,168,169,170,171]
Alginate/nano-clays Mnt and CNC from MCC	- Decrease water solubility; - Increase surface hydrophobicity with CNC and decrease of this parameter with nanoclay addition; - Reduction in WVP; - Tensile properties improved.	Food packaging.	[15]
Alginate with additional compounds of LEO or OEO	- The lower capacity for scavenging ABTS free radicals or quenching singlet oxygen; - The coatings with the essential orange oil are very efficient for controlling yeast and mold growth.	Applying coatings on fresh and cut fruits and vegetables.	[61]
Alginate with additional compounds of OO	- Coatings decrease DR, WL, and total sugars and increase the level of antioxidants; - The delayed activity of PG, PL, and PME was noticed in coated fruit representing the reduced softening and ripening process.	Applying coatings on fresh and cut fruits and vegetables.	[62]
Alginate with additional compounds of tea polyphenols	- Coatings decrease red indices, TCC, RR, electrolyte leakage, and malonaldehyde content and maintain the AAC, TPC, and the activities of antioxidant enzymes while have no significant effect on firmness.	Applying coatings on fresh and cut fruits and vegetables.	[18]
Alginate with additional compounds of *Ficus hirta* fruit extract	- The DR, WL, RR, and MDA content is much lower in the coated samples; - The coating treatment enhances the activities of antioxidant and defense-related enzymes such as SOD, CAT, CHI, GLU, and PAL and the accumulation of phenolic compounds.	Applying coatings on fresh and cut fruits and vegetables.	[172]
Alginate with additional compounds of GSE or GEO	- Coatings reduce WL, maintain firmness during storage, preserve the antioxidant activity of treated grapes, and decrease DR in inoculated fruit.	Applying coatings on fresh and cut fruits and vegetables.	[173]
Sodium Alginate with GP and garlic oil additives	- Casting method and film physical form.	Antibacterial food applications.	[24]
Sodium alginate with calcium chloride additives	- Sprayer methods and coating physical form.	Food protection.	[63]

CNC—cellulose nanocrystals; MCC—microcrystalline cellulose; WVT—water vapor transmission; LEO—lemon essential oil; OEO—orange essential oil; ATBS—acetyltributyl citrate; OO—olive oil; DR—decay rate; WL—weight loss; PG—polygalacturonase; PL—pectate lyase; PME—pectin methyl esterase; TCC—total chlorophylls content; RR—respiration rate; AAC—ascorbic acid content; TPC—total phenolic content; DR—decay rate; MDA—maleicdialdehyde; SOD—superoxide dismutase; CAT—catalase; CHI—chitinase; GLU—β–1,3–glucanase; PAL—phenylalanine ammonia lyase; GSE—grapefruit seed extract; GEO—grapefruit essential oil; GP—glycerol plasticizer.

**Table 3:** Pectin properties used for different applications.

Composite Material	Effect	Possible Application	Reference
Pectin PEG Halloysite nanotubes	- Decrease wettability; - Improve mechanical properties.	Coatings for food conservation.	[14]
Pectin with additional compounds of AAC, CAC and SC	- Coatings reduce microbial spoilage; - They do not significantly influence sensory and nutritional qualities.	Applying coatings on fresh and cut fruits and vegetables.	[73]
Pectin with additional compounds of citral and eugenol	- Coatings are not cytotoxic and do not considerably change the general physicochemical and nutritional characteristics of raspberries; - The impact is mainly on decreasing food spoilage microorganisms and accordingly extending shelf-life.	Applying coatings on fresh and cut fruits and vegetables.	[17]
Pectin with additional compounds of OEO	- Coatings with OEO exhibit antifungal influence on inoculated tomatoes; - Increase TPC and AA; - The sensorial acceptability of the coated tomatoes is well accepted by panelists.	Applying coatings on fresh and cut fruits and vegetables.	[72]
Pectin with additional compounds of OPEO	- Coatings reduce the quality loss and improve the sensory scores during storage; - Nano emulsion-based nano coatings containing essential oil have been effective in bacterial and fungal inactivation.	Applying coatings on fresh and cut fruits and vegetables.	[213]
Pectin–gelatin with GP	Crosslinking than air drying method and film physical form.	Biomedical product.	[22]

PEG—polyethylen glycol; AAC—ascorbic acid; CAC—citric acid; SC—sodium chlorite; OEO—oregano essential oil; TPC—total phenolic content; AA—antioxidant activity; OPEO—orange peel essential oil; GP—glycerol plasticizer.
